# Circadian control of immune homeostasis in cardiovascular health and disease

**DOI:** 10.3389/fimmu.2026.1867223

**Published:** 2026-06-19

**Authors:** Zhaoshan Zhang, Fengmei Zhang, Yuhua Cai, Jiawei Guo

**Affiliations:** 1 Department of Cardiology, The First Affiliated Hospital of Yangtze University, Jingzhou, China; 2 Department of Pharmacology, School of Medicine, Yangtze University, Jingzhou, China

**Keywords:** bmal1, cardiovascular disease, Circadian clock, Macrophage polarization, REV-ERBα, vascular inflammation

## Abstract

The circadian system is an important regulator of cardiovascular immune homeostasis. Emerging evidence suggests that daily timing of immune responses may influence cardiovascular disease progression by coordinating leukocyte trafficking, inflammatory thresholds, metabolic adaptation, and tissue repair across the 24-hour cycle. This review examines how core and auxiliary circadian regulators, including BMAL1, CLOCK, PER/CRY complexes, REV-ERBs, RORs, and systemic timing cues, shape immune-cell activation through transcriptional, epigenetic, metabolic, and neuroendocrine mechanisms. We further synthesize evidence on circadian coordination of leukocyte trafficking, particularly the CXCL12/CXCR4 axis, and discuss how disrupted timing may promote inappropriate leukocyte recruitment into the vascular wall. At the cellular level, circadian misalignment has been associated with altered macrophage polarization, inflammasome activation, and inflammatory injury, processes that may modulate atherosclerosis, myocardial ischemia-reperfusion injury, and post-infarction remodeling. Finally, we evaluate the translational potential and current limitations of chronopharmacology, emphasizing that time-of-day treatment strategies require careful consideration of clinical evidence, circadian phase assessment, chronotype, sex, age, comorbidities, and treatment feasibility. This evidence-weighted chrono-immunological perspective may help refine future research on cardiovascular inflammation and inform the development of more individualized prevention and therapeutic strategies.

## 1 Introduction

Cardiovascular diseases (CVDs) persist as the leading cause of global mortality, characterized by a complex pathophysiology that manifests not only through multi-organ spatial interactions but also via dynamic temporal evolution (1). Over the past several decades, chronic inflammation has been crystallized as a fundamental driver of core pathological processes, including atherosclerosis, myocardial infarction, and heart failure (2). Although anti-inflammatory strategies—most notably those targeting the IL-1β pathway—have demonstrated their efficacy in reducing residual cardiovascular risk in major clinical trials, a critical scientific paradox remains: why do acute events, such as plaque rupture and malignant arrhythmias, exhibit a starkly disproportionate incidence during the early morning hours despite systemic therapeutic intervention (3, 4)? This temporal heterogeneity suggests that the cardiovascular immune microenvironment is not in a static equilibrium but is under the rigorous governance of the endogenous circadian timing system.

The circadian system, composed of the master clock in the suprachiasmatic nucleus (SCN) and autonomous molecular oscillators in peripheral tissues (e.g., vascular endothelium, cardiomyocytes, and immune cells), synchronizes physiological homeostasis with the 24-hour light-dark cycle via integrated transcription-translation feedback loops (TTFL) (5, 6). Emerging evidence in “chrono-immunology” has revealed that nearly every facet of the immune response—ranging from the mobilization of hematopoietic stem cells in the bone marrow and the transendothelial migration of circulating leukocytes to the phenotypic polarization of macrophages—exhibits distinct 24-hour oscillation patterns (7, 8). However, this evolutionary adaptation, designed to anticipate environmental fluctuations, is increasingly compromised by modern lifestyle factors such as light pollution, chronic sleep fragmentation, and shift work. These disruptions may impair cardiac reparative capacity and exacerbate inflammatory signaling in certain experimental and clinical contexts, thereby increasing cardiovascular vulnerability rather than uniformly causing vascular injury (9).

While the individual contributions of circadian biology and immunity to cardiovascular health have been widely investigated, their integration requires a structure that separates basic timing biology, immune rhythmicity, disease-specific manifestations, and translational opportunities. This review therefore organizes the field into distinct mechanistic and clinical layers. Section 2 defines the molecular and systemic architecture of circadian timing. Section 3 focuses on immune-cell rhythmicity, including leukocyte trafficking, innate and adaptive immune timing, and immunometabolic regulation. Section 4 evaluates cardiovascular disease contexts in which circadian disruption may be relevant, emphasizing disease-specific mechanisms rather than a uniform injury pathway. Section 5 examines selected immune mechanisms that may connect disrupted timing to vascular and myocardial pathology. Section 6 discusses chronopharmacology and its clinical constraints, followed by a dedicated limitations section addressing methodological and translational barriers.

### 1.1 Literature search and evidence synthesis

This article was prepared as a narrative review using a structured literature search and evidence synthesis strategy. Relevant studies were identified through searches of PubMed, Web of Science, and Scopus for articles published up to April 2026. Search terms included combinations of “circadian rhythm,” “molecular clock,” “clock-controlled genes,” “BMAL1,” “CLOCK,” “REV-ERBα,” “PER,” “CRY,” “immune homeostasis,” “chrono-immunology,” “leukocyte trafficking,” “macrophage polarization,” “immunometabolism,” “vascular inflammation,” “atherosclerosis,” “myocardial infarction,” “thrombosis,” “arrhythmia,” “heart failure,” “cardiovascular disease,” and “chronotherapy.” Additional studies were identified by screening the reference lists of highly relevant original articles and authoritative reviews.

Studies were considered for inclusion when they directly addressed circadian regulation of immune function, cardiovascular physiology or pathology, inflammatory signaling, leukocyte trafficking, immunometabolic regulation, or time-of-day-dependent therapeutic responses. Priority was given to primary mechanistic studies, well-controlled preclinical models, human observational studies, and clinical intervention studies that provided direct evidence related to circadian, immune, or cardiovascular endpoints. Review articles were used mainly to provide background context and to identify relevant primary literature, rather than as the sole basis for specific mechanistic or clinical claims. Studies were not emphasized when their findings were only indirectly related to circadian biology, immune regulation, or cardiovascular outcomes, or when the experimental context could not be clearly linked to the scope of this review.

Because the available literature spans molecular biology, animal models, epidemiology, and clinical therapeutics, evidence was interpreted according to study type and translational relevance. Mechanistic and cell-specific studies were used to define molecular pathways and candidate regulatory nodes. Preclinical studies were interpreted as model-dependent evidence, particularly when findings were derived from genetic clock disruption, simulated circadian misalignment, or nocturnal rodent models. Human epidemiological studies were treated primarily as associative, especially when sleep duration, shift work, diet, socioeconomic status, comorbidities, occupational stress, or medication timing could act as confounding factors. Clinical trials and intervention studies were weighted most heavily when evaluating chronopharmacology and therapeutic feasibility. This evidence-weighted approach was used to distinguish established mechanisms from model-dependent observations and emerging hypotheses, thereby providing a balanced synthesis of circadian immune regulation in cardiovascular health and disease.

## 2 Circadian rhythm basics: from molecular oscillators to systemic integration

**Figure 1:**
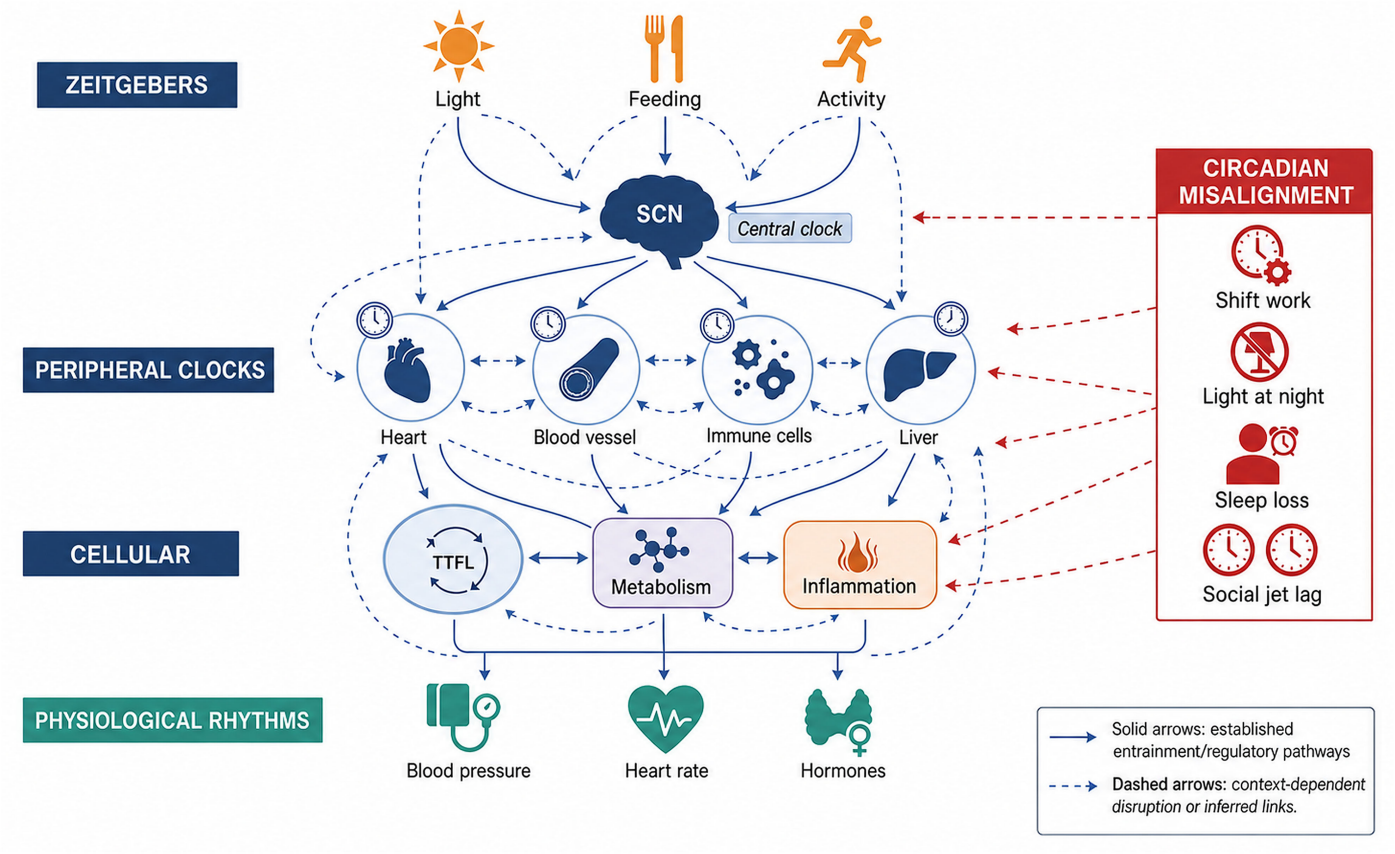
Hierarchical organization and disruption of the mammalian circadian system. The mammalian circadian system is organized in a hierarchical manner, with environmental cues (zeitgebers), such as light, feeding, and physical activity, synchronizing the central clock located in the suprachiasmatic nucleus (SCN). The SCN coordinates peripheral clocks across multiple tissues, including the cardiovascular and immune systems, through neural and hormonal signaling pathways. At the cellular level, circadian rhythms are generated by a transcription–translation feedback loop (TTFL), in which BMAL1/CLOCK drive the expression of PER and CRY proteins that, in turn, inhibit their own transcription. This molecular oscillator regulates a wide range of downstream processes, including metabolism and inflammatory signaling, ultimately contributing to daily variations in physiological functions such as blood pressure and heart rate. Disruption of circadian alignment, as observed in shift work, light exposure at night, or sleep loss, can uncouple central and peripheral clocks and destabilize cellular oscillations, leading to systemic desynchrony. Importantly, this framework should be interpreted as a network-based model in which central pacemaker signals, tissue-specific clocks, neuroendocrine cues, feeding and activity cycles, and local cardiovascular stressors jointly shape immune and cardiovascular rhythms, rather than as a linear pathway controlled by BMAL1 or REV-ERBα alone.

The circadian system represents an evolutionarily conserved timing mechanism that synchronizes internal physiological processes with the external 24-hour light-dark cycle (10). In mammals, this system is organized as a sophisticated hierarchical network, integrating cell-autonomous molecular clocks with a central pacemaker and diverse peripheral oscillators (11). This chapter delineates the fundamental architecture of the circadian machinery, beginning with the intracellular feedback loops that generate rhythmicity, and subsequently exploring the systemic integration of these signals across tissues. A clear understanding of these core biological principles is necessary for interpreting how altered timing may influence immune and cardiovascular regulation in later disease-specific contexts (**Figure 1**).

### 2.1 The intracellular molecular clockwork: the TTFL core

At the cellular level, circadian rhythmicity is orchestrated by an autonomous, evolutionarily conserved transcription-translation feedback loop (TTFL) (12, 13). This molecular engine does not merely serve as a passive timer; rather, it functions as a master rheostat that aligns cellular metabolic flux, proteostasis, and inflammatory signaling with the 24-hour environmental cycle (14) (**Figure 1**). The core oscillator is initiated by the BMAL1: CLOCK transcriptional complex, which binds E-box elements and activates a broad set of clock-controlled genes, including the negative-arm regulators Period (PER1-3) and Cryptochrome (CRY1-2) (15). However, BMAL1: CLOCK should be viewed as one component of a wider regulatory network rather than as a single dominant axis (16). Oscillator stability and tissue-specific output depend on feedback from PER/CRY complexes, nuclear receptors such as REV-ERBs and RORs, chromatin modifiers, metabolic cofactors, and extracellular entrainment signals (17). Thus, the circadian clock operates as an interconnected regulatory system in which transcriptional, epigenetic, metabolic, and environmental inputs jointly shape rhythmic cellular function (18) (**Figure 1**).

The precision of this 24-hour cycle is governed by the progressive accumulation, phosphorylation, and subsequent nuclear translocation of PER and CRY proteins (19). Once a critical nuclear concentration is reached, the PER: CRY complex physically displaces the BMAL1: CLOCK activator from the E-box, or recruits co-repressors like HDACs (histone deacetylases) to silence transcription. This primary loop is further reinforced by an interlocking regulatory network involving the nuclear receptors REV-ERB***β*** and ROR***α***, which compete for binding at ROR response elements (ROREs) within the *Bmal1* promoter (20). While RORs typically stabilize the oscillation by promoting *Bmal1* expression, REV-ERBs function as potent transcriptional repressors (21). REV-ERBs provide one important interface between the molecular clock and immunometabolism by linking intracellular heme availability to transcriptional repression of selected inflammatory and metabolic genes (22).Nevertheless, this regulatory layer acts together with ROR-dependent transcriptional activation, PER/CRY-mediated feedback repression, CLOCK-associated chromatin remodeling, and metabolic sensors such as AMPK, SIRT1, and HIF-1α. Innate immune rhythmicity therefore emerges from multiple converging regulatory inputs rather than from REV-ERB activity alone (23).

Beyond these core components, the TTFL’s influence is amplified through the rhythmic modulation of Clock-Controlled Genes (CCGs), which can account for up to 40% of a tissue’s transcriptome (24). This temporal control extends to the regulation of oxidative stress responses (via the NRF2 pathway), autophagy, and the periodic sensitivity of the vascular endothelium to hemodynamic shear stress (25) (**Figure 1**). The biological significance of this molecular architecture is perhaps most evident in its failure. Genetic ablation of core components, including Bmal1, provides important evidence that cell-autonomous clocks help maintain temporal organization of inflammatory and metabolic pathways (26). However, the consequences of clock-gene disruption are context-dependent and may differ across immune cells, endothelial cells, cardiomyocytes, and vascular smooth muscle cells (27). Rather than representing a simple linear sequence from clock-gene loss to cardiovascular pathology, these findings support a network model in which altered NF-κB signaling, inflammasome activation, redox imbalance, mitochondrial metabolism, and tissue-specific stress responses jointly contribute to disease susceptibility (28, 29).

### 2.2 Hierarchical organization of the mammalian circadian system

The mammalian circadian system exhibits a sophisticated hierarchical architecture designed to transduce environmental cues into stable physiological rhythms (30, 31). The suprachiasmatic nucleus (SCN) serves as the “master pacemaker,” receiving photic input via the retinohypothalamic tract (RHT) from melanopsin-containing intrinsically photosensitive retinal ganglion cells (ipRGCs) (31, 32). This specialized neural pathway enables the SCN to sense the external light/dark cycle and subsequently reset internal biological time, ensuring precise synchronization with the Earth’s rotation (30).

The systemic integrity of this network relies on the diverse peripheral clocks distributed throughout various organs (33). Within the cardiovascular context, cardiomyocytes, endothelial cells, and bone marrow-derived immune cells possess these autonomous timing mechanisms (34). Unlike the SCN, which prioritizes environmental sensing, these peripheral oscillators are primarily dedicated to functional execution; for instance, the cardiac clock modulates myocardial oxygen utilization while the endothelial clock governs the timing of vasodilatory factor release (35). To prevent internal desynchrony, the SCN orchestrates these peripheral organs through the autonomic nervous system (ANS) and endocrine pathways. SCN-derived signals are projected via sympathetic and parasympathetic fibers to regulate catecholamine release, driving diurnal fluctuations in heart rate and leukocyte mobilization (36). In addition to catecholaminergic signaling, parasympathetic cholinergic pathways also provide an important neuroimmune interface (37). Vagal efferent activity can modulate immune responses through acetylcholine-dependent signaling, often referred to as the cholinergic anti-inflammatory pathway (38). In this circuit, acetylcholine-sensitive mechanisms, including α7 nicotinic acetylcholine receptor signaling on macrophages and other immune cells, can restrain excessive production of pro-inflammatory cytokines such as TNF-α and IL-1β (39). Because vagal tone, heart rate variability, sleep–wake state, and inflammatory responsiveness all exhibit daily variation, cholinergic signaling may contribute to the temporal coordination of immune tone as well as cardiovascular autonomic balance (40). Thus, autonomic entrainment of peripheral clocks should be understood as involving both sympathetic catecholaminergic outputs and parasympathetic acetylcholine-mediated immunomodulation. Concurrently, the SCN orchestrates the rhythmic secretion of glucocorticoids by modulating the hypothalamic-pituitary-adrenal axis (HPA axis) (35). These systemic hormonal pulses act as potent entrainment signals that unify the phase of peripheral oscillations (41, 42). Such multi-dimensional coupling ensures that the cardiovascular and immune systems maintain homeostatic synergy when responding to metabolic demands.

This hierarchical organization also argues against a two-node interpretation of circadian cardiovascular regulation. Central pacemaker signals, glucocorticoid rhythms, autonomic tone, feeding cycles, tissue-specific clocks, endothelial shear stress, and mitochondrial metabolism jointly determine how circadian information is translated into immune and cardiovascular outputs (43). Accordingly, BMAL1 and REV-ERBα are best understood as representative nodes within a broader timing network rather than as isolated master switches (44).

### 2.3 Zeitgebers and the plasticity of circadian entrainment

The systemic stability of the circadian network relies not only on the robustness of endogenous oscillations but also on its remarkable capacity to integrate and adapt to environmental cues, known as zeitgebers (45). This regulatory plasticity allows the organism to dynamically reshape its physiological rhythms in alignment with behavioral and environmental shifts (46). While light remains the dominant factor for maintaining central rhythmicity, peripheral oscillators within the cardiovascular and immune systems exhibit profound dependency on non-photic signals (47). Among these, feeding cues have emerged as the most potent secondary zeitgebers for modulating the phase of peripheral clocks (48). Regular nutritional intake can independently restructure the metabolic landscape of the heart and vascular wall by activating nutrient-sensing pathways, including insulin signaling and adenosine monophosphate-activated protein kinase (AMPK) (49) (**Figure 1**). This behavioral remodeling is highly tissue-specific; when nutrient timing conflicts with the light/dark cycle (e.g., nocturnal feeding), peripheral clocks rapidly diverge from SCN orchestration, precipitating a state of internal desynchrony (50). Such misalignment not only impairs the metabolic flexibility of cardiomyocytes but also triggers chronic inflammatory responses within the endothelium (51).

Beyond metabolic signals, exercise functions as a critical behavioral zeitgeber that reinforces the rhythmic amplitude of the cardiovascular system (52). Regular physical activity synchronizes peripheral clocks through multiple conduits, including periodic shifts in core body temperature, stimulation of sympathetic tonus, and the rhythmic release of myokines (53). This behavioral plasticity demonstrates that the circadian machinery is not a hardwired program but a dynamic, targetable network, providing a mechanistic foundation for “chrono-interventions” aimed at restoring compromised biological timing (54).

The precise calibration of molecular oscillators by zeitgebers is ultimately manifested in the temporal niche of physiological parameters (55). Under homeostatic conditions, core physiological indices exhibit highly predictable diurnal fluctuations designed to provide metabolic redundancy for the active phase (56). For instance, driven by the rhythmic activity of the sympathetic nervous system and the renin-angiotensin system, blood pressure (BP) typically undergoes a marked physiological elevation upon awakening, termed the morning surge (56)(**Figure 1**). Concurrently, heart rate (HR) and systemic cortisol levels reach their zenith to prime the organism for imminent physiological demands (57). However, these evolutionarily advantageous peaks transform into a “window of vulnerability” under pathological conditions. The early morning surge in catecholamines and cortisol, while enhancing cardiac output, acutely elevates vascular shear stress and promotes pro-thrombotic platelet aggregation (58). The convergence of these physiological stressors may help explain the disproportionate incidence of acute myocardial infarction, aortic dissection, and sudden cardiac death during the early morning hours (59). This temporal clustering of adverse events not only highlights the intrinsic rhythmicity of disease but also underscores the urgency of integrating chronobiology into cardiovascular risk assessment (60).

Terminology for cross-species phase translation. Because much of the experimental literature in circadian immunology is derived from nocturnal rodents, careful phase terminology is required when translating findings to humans (47). Under standard laboratory lighting conditions, Zeitgeber Time 0 (ZT0) usually denotes lights on and ZT12 denotes lights off (61). For nocturnal rodents, the light phase, approximately ZT0–ZT12, corresponds mainly to the rest phase, whereas the dark phase, approximately ZT12–ZT24, corresponds mainly to the active phase (62). In diurnal humans, this relationship is functionally inverted: daytime usually corresponds to the active phase and nighttime to the rest phase (63). Therefore, rodent ZT should not be translated directly into the same human clock time. Throughout this review, cross-species comparisons are interpreted primarily according to biological phase, namely rest phase versus active phase, rather than by numerical ZT alone. When relevant, tables distinguish human clock time, rodent ZT, rest/active phase, and the direction of translation.

### 2.4 Circadian misalignment: definitions, exposures, and pathophysiological associations

To avoid conflating biologically related but non-identical exposures, it is necessary to distinguish cell-intrinsic clock disruption, behavioral circadian misalignment, and sleep loss. Cell-intrinsic clock disruption refers to altered transcription–translation feedback loops or clock-controlled gene programs within specific cell types, including immune cells, endothelial cells, vascular smooth muscle cells, and cardiomyocytes (27, 64). Behavioral circadian misalignment refers to a mismatch between endogenous biological timing and external or behavioral cycles, as occurs with shift work, irregular feeding, light exposure at night, or social jetlag (65). Sleep loss, in contrast, refers primarily to reduced sleep duration or impaired sleep continuity (66). Although these conditions may coexist and interact biologically, they are not interchangeable. Cell-intrinsic clock disruption provides mechanistic insight into molecular and cellular pathways, whereas behavioral misalignment and sleep loss in human populations are often accompanied by confounding factors such as diet, occupational stress, socioeconomic status, obesity, hypertension, comorbidities, and medication timing (67). Therefore, evidence linking circadian disruption to cardiovascular outcomes should be interpreted according to the experimental context and level of causal inference (**Figure 1**).

In modern society, technological advancements have fundamentally reshaped the temporal ecology of human existence, leading to widespread circadian misalignment between endogenous biological clocks and external environmental cycles (68). This dysrhythmia typically originates from external desynchrony, where individuals are exposed to irregular light-dark cycles or non-physiological behavioral patterns (69). Shift work represents the most severe form of this disruption, forcing the organism to engage in feeding and activity during biological sleep phases, thereby inducing a profound conflict between light signals perceived by the SCN and metabolic cues received by peripheral organs (70). Furthermore, social jetlag—the discrepancy in sleep timing between work days and free days—and the ubiquity of artificial light at night (ALAN) further erode the homeostatic thresholds of the circadian system (71) (**Figure 1**). The persistence of these environmental stressors eventually precipitates internal desynchrony, uncoupling systemic hemodynamic peaks from their necessary metabolic support windows within the cardiovascular system (72) (**Figure 1**).

At a deeper mechanistic level, circadian disruption may involve coordinated alterations in redox balance, mitochondrial function, proteostasis, and epigenetic regulation (73, 74). However, the extent to which these processes operate in human cardiovascular disease depends on the type of circadian exposure, tissue context, species background, and supporting evidence (75). When the TTFL loses its synchrony, cellular oxidative stress levels escalate significantly due to the failure of time-gated antioxidant enzyme systems (76). The breakdown of redox regulation by the molecular clock leads to the excessive accumulation of reactive oxygen species during inactive phases, which in turn impairs mitochondrial respiratory chains and destabilizes proteostasis (77). This micro-level imbalance triggers protein misfolding and activates endoplasmic reticulum stress (ER stress) signaling pathways (78, 79). Critically, circadian misalignment induces profound epigenetic remodeling; by altering the diurnal dynamics of histone acetylation or DNA methylation, it facilitates the constitutive transcription of pro-inflammatory genes that are normally repressed (80).

Within the cardio-immunological landscape, circadian misalignment may create a permissive environment for vascular inflammation by altering endothelial barrier function, leukocyte activation, and immune-metabolic balance (44). In experimental models, these alterations have been linked to plaque progression, blood pressure dysregulation, and adverse remodeling (81). In human populations, however, such associations should be interpreted cautiously because behavioral circadian disruption and sleep loss frequently coexist with lifestyle, metabolic, occupational, and therapeutic confounders (**Figure 1**). Thus, circadian misalignment should be viewed as a context-dependent modifier of cardiovascular vulnerability rather than as a universal or isolated causal driver.

## 3 Circadian regulation of the immune system

The immune system is not a static defensive network in a state of constant, uniform alertness but a dynamic, oscillating system integrated across the temporal axis. This regulation fundamentally addresses the “resource allocation paradox” of immunosurveillance: the organism cannot sustain maximal defensive intensity across all tissues at all times (6). Consequently, the immune system has evolved a sophisticated strategy of temporal compartmentalization, utilizing molecular clocks to synchronize energetically demanding processes—such as leukocyte trafficking, cytokine release, and antigen presentation—with the organism’s metabolic cycles (82). Preceding the onset of the active phase, the system preemptively deploys immune forces to potential injury sites to maximize defensive efficacy during peak pathogen exposure (83). This systemic coordination is orchestrated not only by central neuroendocrine axes but also by cell-intrinsic clocks that exert epigenetic control over genomic accessibility. This chapter adopts a compartment-based framework, moving from bone marrow hematopoietic niches and circulating leukocytes to vascular endothelium, plaque microenvironments, myocardium, and systemic neuroendocrine cues, to illustrate how circadian immune regulation emerges from interactions among multiple tissues rather than from a single clock-gene axis.

### 3.1 Rhythmic trafficking and distribution of immune cells

The defensive efficacy of the immune system does not rely on a uniform distribution but on anticipatory trafficking governed by circadian rhythms (84). Under homeostatic conditions, the total leukocyte count in peripheral blood exhibits significant diurnal fluctuations, essentially reflecting the dynamic redistribution of immune cells between the circulation and various organs such as the bone marrow, spleen, lungs, and lymph nodes (85). For most myeloid subsets, peak blood concentrations typically occur during the resting phase and decline sharply before the onset of the active phase. This decline is not a manifestation of cell loss but reflects a strategic infiltration into peripheral tissues to preemptively address the increased risk of infection or trauma associated with active behavior.

This complex migratory process is co-driven by the sympathetic nervous system (SNS) and the cell-intrinsic molecular clocks (82, 86). The bone marrow serves as a reservoir, where the release and sequestration of leukocytes are primarily governed by the CXCL12 (Stromal cell-derived factor 1) and CXCR4 signaling axis (87). Rhythmic release of norepinephrine from SNS terminals within the bone marrow acts on ***β***-adrenergic receptors of stromal cells, thereby suppressing *CXCL12* transcription (88) (**Table 1**). This periodic decline in chemokine gradients drives the rhythmic mobilization of hematopoietic stem cells and mature leukocytes into the peripheral blood (89). Simultaneously, the expression of adhesion molecules on the vascular endothelial surface exhibits high temporal specificity. For instance, the expression of Vascular cell adhesion molecule 1 (VCAM-1) and Intercellular adhesion molecule 1 (ICAM-1) follows distinct phases across different organs (90). This spatiotemporal gating mechanism contributes to organ-specific immune-cell homing within defined temporal windows.

**Table 1:** Spatiotemporal landscape of circadian immune cell trafficking.

Immune cell	Human clock time/phase		Rodent ZT/phase	Direction of cross-species translation	Key molecular drivers		Mechanism of tissue redistribution	Cardiovascular pathophysiological implications	References
Neutrophils	Night to early morning; late rest-to-wake transition		ZT5–ZT13; late light phase to early dark transition; rest-to-active transition in nocturnal rodents	Translate by biological phase rather than identical clock time; rodent ZT12 approximates active-phase onset in rodents, not human evening	CXCL12/CXCR4; CXCL1/CXCR2		Circadian aging leads to CXCR4 upregulation and rhythmic clearance in bone marrow/spleen; CXCL1-driven recruitment increases around the active-phase transition	May contribute to time-of-day variation in myocardial ischemia-reperfusion injury and microvascular plugging	(99)
Monocytes		Early morning or wake transition; timing varies by study and compartment	ZT8–ZT12; late light/rest phase before active-phase onset	Compare rodent late-rest phase with human pre-wake or early-wake biology, not identical clock hours	CCL2; CCR2; VCAM-1		Rhythmic endothelial adhesion molecule expression and chemokine gradients regulate monocyte recruitment into vascular tissues	May influence temporal variation in plaque inflammation and monocyte recruitment	(100)
NK Cells		Daytime or active phase, context-dependent	ZT13–ZT21; dark/active phase in nocturnal rodents	Translate as active-phase immune surveillance rather than direct evening equivalence in humans	CX3CL1; CX3CR1		Adrenergic signaling and tissue chemokine gradients regulate time-of-day variation in cytotoxic activity and tissue infiltration	May participate in vascular surveillance and viral myocarditis defense	(11)
T Lymphocytes		Night or early rest phase, depending on subset and lymphoid compartment	ZT1–ZT5; early light/rest phase in nocturnal rodent	Interpret as rest-phase lymphoid redistribution; direct human clock-time equivalence is not appropriate	S1P1; L-selectin		SNS-driven redistribution to lymph nodes; S1P1 regulates rhythmic lymphoid egress	May modulate adaptive immune responses in post-infarction remodeling and vasculitis	(101)
B Lymphocytes		Night or late rest phase; subset-dependent	ZT9–ZT13; late light/rest-to-active transition	Translate according to rest-to-active transition and lymphoid tissue context	CXCL13; CXCR5		Clock-associated chemotaxis toward lymphoid organs contributes to rhythmic B-cell positioning	May influence antibody-mediated vascular inflammation and IL-10-associated atheroprotection	(102)
Tregs		Late daytime to evening or post-active phase, context-dependent	ZT14–ZT18; early-to-mid dark/active phase in nocturnal rodents	Translate as active-phase or post-activation immune restraint rather than identical clock time		CCR4; CCL22	Phase-shifted from effector T cells to restrain excessive inflammatory responses	Supports cardiovascular immune homeostasis and may limit adverse chronic remodeling after MI	(103)
HSCs		Morning to daytime mobilization reported in some human studies; context-dependent	ZT13–ZT17; early dark/active phase in nocturnal rodents	Translate as active-phase-associated mobilization while considering species-specific sympathetic and glucocorticoid rhythms		β3-adrenergic signaling; CXCL12	Rhythmic norepinephrine release suppresses Cxcl12 in bone marrow niches, promoting systemic mobilization	May sustain myeloid progenitor supply during chronic vascular inflammation	(81)
Eosinophils		Night or rest phase, tissue-dependent	ZT8–ZT12; late light/rest phase in nocturnal rodents	Interpret according to rest-phase tissue recruitment rather than direct human clock time		CCL11; CCR3	Tissue-resident clocks regulate rhythmic eosinophil recruitment in lung and gastrointestinal endothelium	Relevant to eosinophilic myocarditis and asthma-related cardiovascular complications	(104)

The systemic distribution of immune cells is profoundly regulated by the epigenetic remodeling of the tissue microenvironment (91). Recent findings suggest that tissue-resident cells utilize molecular clocks to recruit chromatin modifiers, thereby modulating the chromatin accessibility of enhancer regions for chemokine genes (92). For instance, endothelial cells in the lung utilize their circadian machinery to periodically recruit histone deacetylase 3 (HDAC3) (93) (**Table 1**). At specific phases, HDAC3-mediated deacetylation restricts the acetylation levels of the CCL2 (Monocyte chemoattractant protein-1) promoter, effectively “closing” the gene; in opposite phases, the chromatin shifts to an open state, permitting the massive transcriptional burst of CCL2 (93)(**Table 1**). This epigenetic temporal gating may help restrict monocyte infiltration to specific phases under homeostatic conditions.

At the single-cell level, intrinsic molecular clocks endow leukocytes with autonomous migratory rhythms by coupling cytoskeletal dynamics with metabolic flux (94). BMAL1 has been implicated in the rhythmic regulation of cytoskeletal programs, including Rho GTPase family members such as RhoA and Cdc42, which participate in cellular polarization and pseudopodia formation (95). The rhythmic expression of these proteins grants leukocytes enhanced deformability and endothelial transvasation capacity during the active phase (96). This migratory rhythm is intrinsically linked to immunometabolism; during peak trafficking periods, the molecular clock upregulates glycolytic enzyme activities to provide the ATP bursts required for rapid cytoskeletal reorganization (97). Neutrophils exhibit a unique “circadian aging” phenotype. As they age within the circulation, they rhythmically upregulate surface CXCR4 expression while simultaneously decreasing levels of PSGL-1 (P-selectin glycoprotein ligand-1) (47)(**Table 1**). This shift in the surface receptor repertoire causes “aged” neutrophils to lose their recruitment capacity toward inflammatory sites, redirecting them toward the bone marrow and spleen via the CXCL12 gradient (87). Within these niches, macrophages eliminate them through efferocytosis, a process that triggers the activation of the transcription factor LXR (Liver X receptor) (61) (**Table 1**). This, in turn, suppresses the production of IL-23 in the bone marrow. This negative feedback loop, mediated by cell clearance signals, supports homeostatic renewal of circulating leukocytes and helps prevent excessive inflammation during inactive phases by dampening systemic pro-inflammatory cytokine levels (98). These trafficking rhythms provide a biological basis for time-of-day variation in leukocyte availability and tissue recruitment. Their disease-specific relevance is discussed below in relation to vascular inflammation, thrombosis, and myocardial injury (75). Because rodents are nocturnal whereas humans are diurnal, the following table separates rodent ZT from human clock time and emphasizes biological phase translation. Rodent ZT intervals should be interpreted according to rest or active phase rather than directly mapped onto identical human clock hours (**Table 1**).

### 3.2 Temporal gating of innate immune responses

The innate immune system, serving as the primary line of defense against pathogens and systemic stressors, exhibits profound circadian characteristics (11). The core of this “temporal gating” mechanism lies in the precise regulation of the activation threshold and effector intensity of innate immune responses, ensuring peak sensitivity during high-risk periods (105). This orchestration begins with the rhythmic expression of pattern recognition receptors (PRRs). Notably, Toll-like receptor 9 (TLR9) is a quintessential example of clock-gated regulation; the core clock protein BMAL1 directly binds to E-box elements within its promoter, driving robust diurnal oscillations in transcription (106, 107). Consequently, identical ligand concentrations elicit a significantly more potent immune response during the active phase than during the resting phase. Furthermore, TLR4-mediated endotoxin responses are finely tuned by the molecular clock, which modulates the availability of the adapter protein MYD88 and the nuclear translocation efficiency of NF-κB, thereby establishing specific temporal windows for the burst release of pro-inflammatory cytokines such as TNF-A and IL-6 (108, 109).

Downstream of PRR signaling, NLRP3 inflammasome activity is shaped by multiple circadian and metabolic regulators. REV-ERBα can repress selected inflammatory genes, including *NLRP3* and *IL1b*, but inflammasome rhythmicity also depends on NF-κB priming, mitochondrial redox state, metabolic substrate availability, glucocorticoid signaling, and cell-type-specific clock outputs (110). This inhibition peaks during the resting phase, shielding the organism from spontaneous inflammatory responses induced by oxidative stress or metabolic byproducts (22). When this temporal constraint is compromised due to circadian misalignment or clock gene deficiency, the asynchronous overactivation of the NLRP3 inflammasome leads to the excessive spillover of IL-1B (17). In cardiovascular tissues, this imbalance may contribute to endothelial inflammatory signaling and plaque immune activation, although disease-specific interpretation requires consideration of local vascular context and supporting evidence (111).

**Figure 2:**
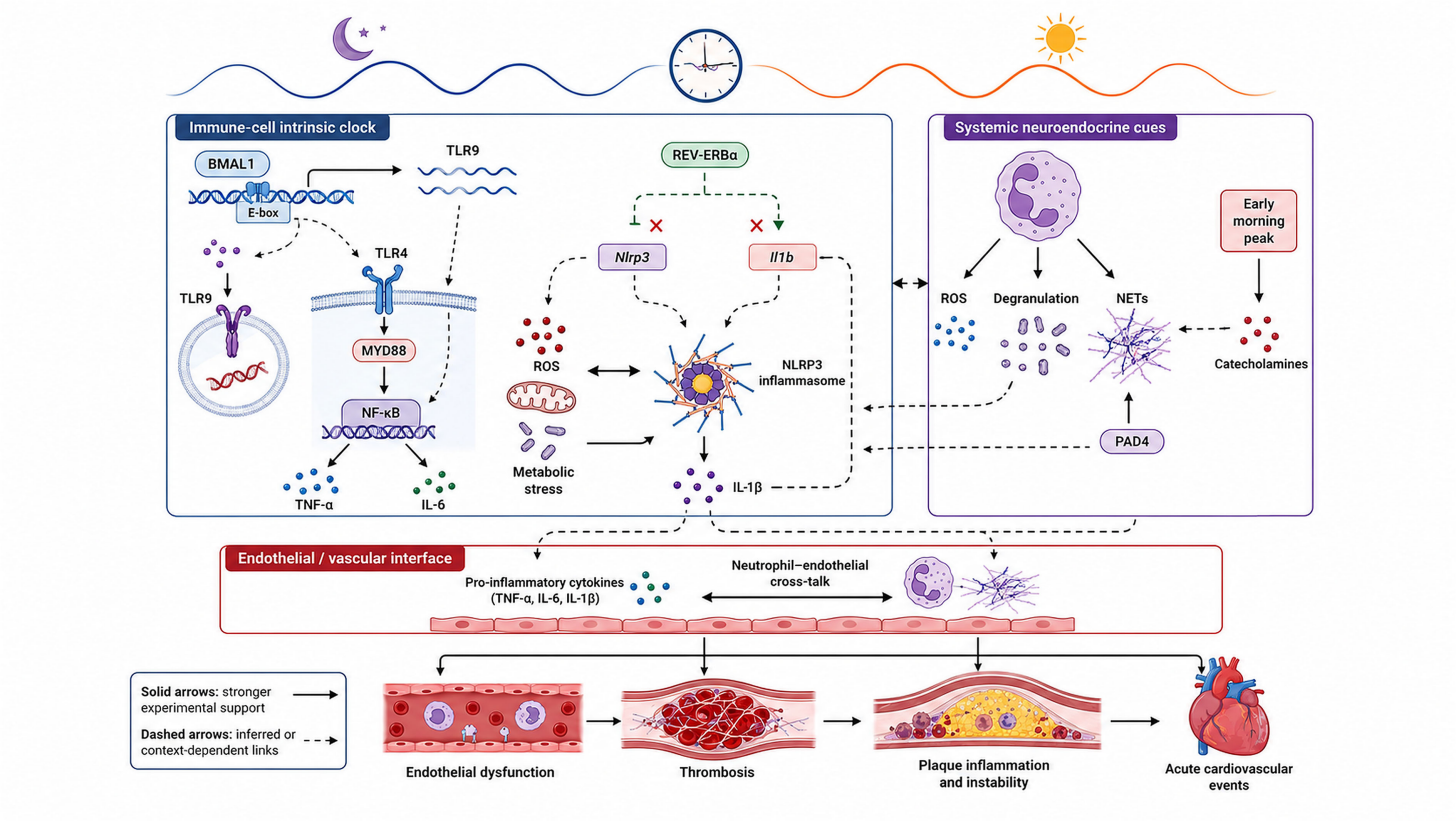
Circadian control of innate immune activation and its vascular consequences. Circadian mechanisms impose temporal constraints on innate immune activation at multiple levels. Core clock components regulate the sensitivity of pattern recognition receptor signaling, including TLR pathways, and modulate downstream inflammatory outputs through factors such as NF-κB. In parallel, negative regulation of the NLRP3 inflammasome by REV-ERBα limits excessive IL-1β production. Effector functions of neutrophils, particularly NETs formation, also exhibit time-of-day variation. Disruption of this temporal organization may enhance pro-inflammatory signaling and contribute to endothelial dysfunction and thrombosis in susceptible cardiovascular contexts.

Beyond the gating of receptors and signaling molecules, the functional outputs of innate effector cells also display clear diurnal rhythms (**Figure 2**). As frontline effectors, neutrophils exhibit killing capacities—including the generation of reactive oxygen species (ROS) and degranulation—that are strictly constrained by endogenous clocks (112). Of profound clinical significance, the capacity for neutrophil extracellular traps (NETs) formation peaks during the early morning hours, coinciding with the transition to the active phase (113). This process is driven by the clock-controlled expression of peptidylarginine deiminase 4 (PAD4) (47). When triggered by the early-morning surge in catecholamines, this heightened capacity for NETs release facilitates pathogen entrapment but simultaneously escalates thrombotic risk and compromises vascular integrity (114). These observations indicate that innate immune effector functions are temporally structured, with different phases associated with distinct inflammatory and thrombotic potentials (**Figure 2**).

### 3.3 Circadian control of adaptive immunity

Unlike the instantaneous defense of innate immunity, the adaptive immune system exhibits a profound temporal compartmentalization, orchestrating antigen recognition, lymphocyte activation, and effector evolution in precise alignment with metabolic rhythms (115). This regulation commences with the circadian gating of antigen presentation by dendritic cells (DCs). As the critical bridge between innate and adaptive responses, DC maturation and trafficking are under strict rhythmic control (116). Specifically, the rhythmic upregulation of the chemokine receptor CCR7 on DCs, typically preceding the active phase, ensures their precise migration from peripheral tissues to draining lymph nodes (117). This “temporal gating” optimizes the efficiency of antigen presentation during windows of peak environmental exposure, initiating downstream T-cell priming with high temporal fidelity (118).

Within the lymphoid microenvironment, T-cell activation and lineage commitment are governed by intrinsic molecular clocks, resulting in distinct phase-dependent profiles (119). Earlier studies often described adaptive immune rhythmicity in terms of Th1/Th2 balance, including time-of-day variation in cytokines such as IFN-γ and IL-4 (120). However, this binary framework does not fully capture the current understanding of T-cell heterogeneity. Circadian regulation may influence a broader spectrum of T-cell states, including Th1-like, Th2-like, Th17, Treg, follicular helper T-cell, memory, exhausted, and tissue-resident programs, depending on antigenic context, tissue microenvironment, metabolic state, and disease stage (121). Therefore, Th1/Th2 terminology is used here only as a historical and simplified reference rather than as a complete representation of adaptive immune programming. Concurrently, the suppressive potency of regulatory T cells (Tregs) peaks during the resting phase. This evolutionary design facilitates the resolution of inflammatory stress accumulated during the active phase, maintaining systemic immunological homeostasis (122). In a cardiovascular context, this temporal heterogeneity in T-cell signatures directly influences inflammatory resolution and tissue remodeling following myocardial infarction (75).

Humoral immunity, centered on B-cell responses and the formation of germinal centers, is similarly subsumed under circadian orchestration (105). B-cell proliferation, somatic hypermutation, and affinity maturation within lymphoid follicles exhibit clear time-dependency (119). The expression of the pivotal enzyme activation-induced cytidine deaminase (AID) is directly controlled by the molecular clock, determining the efficiency of immunoglobulin class-switch recombination and the diversification of the antibody repertoire (123). These rhythmic dynamics have substantial clinical implications, particularly regarding vaccine efficacy; immunization during specific temporal windows—such as the early morning—frequently elicits more robust germinal center reactions and higher antibody titers (124). These findings indicate that adaptive immune responses are also temporally organized, although their relevance to cardiovascular inflammation and therapeutic timing requires disease-specific interpretation (125–127).

### 3.4 Immunometabolic rhythms: the molecular integration

The spatiotemporal dynamics of the immune system are closely linked to underlying metabolic rhythms. The functional transition of immune cells—from steady-state surveillance during resting phases to pro-inflammatory bursts during active phases—relies on a profound shift in energy substrate utilization (128). These immunometabolic rhythms represent a deep integration between molecular clocks and metabolic sensing pathways, ensuring energetic efficiency and redox homeostasis throughout the circadian cycle (129). Within the diurnal period, immune cells exhibit distinct metabolic preferences: during the active phase, to support rapid migration, cytokine synthesis, and phagocytosis, immune cells (particularly myeloid lineages) preferentially upregulate glycolysis (130). This process is influenced by cross-talk among BMAL1-dependent transcriptional programs, HIF-1α activity, nutrient-sensing pathways, mitochondrial function, and cellular redox state (131).Rather than acting through a single BMAL1-HIF-1α switch, immunometabolic rhythmicity reflects the integration of clock-controlled transcription with metabolic feedback loops (132). Conversely, during the resting phase, the metabolic axis shifts toward oxidative phosphorylation (OXPHOS) and fatty acid oxidation. This transition, orchestrated by the AMPK (AMP-activated protein kinase) signaling pathway, minimizes energetic expenditure and promotes tissue repair through the production of anti-inflammatory metabolites such as itaconate (133).

As the structural hubs of immunometabolism, mitochondrial morphology and function are subject to precise circadian orchestration. The molecular clock drives a fusion-fission cycle by regulating the phosphorylation of mitochondrial fusion proteins (Mitofusins) and fission proteins (e.g., DRP1) (134). Mitochondria tend toward a fissioned state during the active phase to optimize ATP bursts, whereas a fused morphology during the resting phase favors electron transport chain efficiency and organelle repair (135). Simultaneously, the endogenous clock activates the NRF2 pathway to enhance antioxidant defenses during metabolic peaks, neutralizing collateral damage from metabolic byproducts such as reactive oxygen species (ROS) (135). These metabolic rhythms serve not only as functional supports but also as sources of circadian feedback. Fluctuations in metabolite concentrations—such as NAD^+^, acetyl-CoA, and ***α***-ketoglutarate (α-KG)—directly modulate the activity of chromatin modifiers, including SIRT1 and various histone acetyltransferases (136). This metabolic-epigenetic coupling links the organism’s nutritional status to the rhythmic transcriptional landscape of immune cells. In cardiovascular pathophysiology, the disruption of this integration—triggered by factors like high-fat diets or chronic sleep deprivation—leads to metabolic phase-shifting (137). Such misalignment reshapes the immunometabolic blueprint, fostering a persistent pro-inflammatory phenotype that underpins the progression of chronic inflammatory vascular diseases like atherosclerosis (138).

## 4 Circadian disruption and cardiovascular disease

This section shifts from general immune rhythmicity to disease-specific cardiovascular contexts. Rather than presenting circadian disruption as a uniform mechanism across all conditions, the following subsections emphasize distinct clinical or pathophysiological dimensions: autonomic and blood pressure timing in hypertension, endothelial barrier function and vascular wall remodeling in atherosclerosis, cardiomyocyte metabolic and mitochondrial vulnerability in myocardial ischemia-reperfusion injury, electrophysiological timing in arrhythmia, and evidence strength across additional cardiovascular conditions.

### 4.1 Hypertension and autonomic dysregulation

The diurnal rhythm of blood pressure (BP) is one of the most fundamental biological hallmarks of cardiovascular stability, and its disruption—clinically identified as circadian misalignment—serves as a prospective indicator for cardiovascular remodeling and organ damage (75). Under physiological conditions, BP exhibits a characteristic “dipping” pattern, where nocturnal BP decreases by 10%–20% relative to daytime levels (139). This process is precisely orchestrated by central oscillators (e.g., the SCN) modulating autonomic nervous tone (139). However, disruption of circadian organization may impair this predictive regulatory machinery and is often associated with autonomic dysregulation (140).The attenuation of SCN output signals or phase shifts in peripheral clocks leads to sustained nocturnal hyperactivation of the sympathetic nervous system (SNS) and a concomitant failure to elevate vagal tone (141). This spatiotemporal mismatch in neural drive may contribute to “non-dipping” blood pressure patterns (142).

From a biomechanical perspective, persistent nocturnal hypertension imposes significant mechanical stress on the vessel wall (143). In the absence of a nocturnal low-pressure “buffering period,” resistance arterioles remain in a chronic state of high tension, leading to compensatory smooth muscle hypertrophy and fibrosis, which eventually culminates in arterial stiffening (144). Furthermore, circadian disruption has been associated with impaired baroreceptor sensitivity in experimental and clinical contexts (145). In a healthy rhythmic state, BRS fluctuates diurnally to buffer sudden BP surges during different behavioral activities (146). However, the loss or dysfunction of core clock genes (e.g., *BMAL1*) blunts the signaling efficiency within the baroreflex arc, stripping the organism of its capacity to regulate acute hemodynamic fluctuations (147). Over time, persistent nocturnal pressure load and impaired vascular tone regulation may contribute to vascular remodeling, increased cardiac afterload, left ventricular hypertrophy, and heart failure risk (35).

### 4.2 Atherosclerosis and vascular wall remodeling

Atherosclerosis involves chronic interactions among endothelial dysfunction, lipid retention, inflammatory cell recruitment, and vascular smooth muscle cell remodeling (148). Circadian disruption may modulate several of these processes, but the available evidence ranges from cell-specific and animal-model data to associative human observations (149). Experimental studies suggest that altered endothelial clocks can impair nitric oxide signaling, barrier integrity, and adhesion molecule expression, thereby creating a vascular environment more permissive to inflammatory cell recruitment (150). The vascular endothelium, acting as the frontline sensor for hemodynamic changes, operates a sophisticated “defense program” driven by core clock genes (151). Under physiological conditions, BMAL1 orchestrates the efficient coupling of endothelial nitric oxide synthase (eNOS) during active phases to produce sufficient nitric oxide (NO), thereby dilating vessels and counteracting the mechanical strain of blood pressure surges (152, 153). In experimental settings of circadian disruption, eNOS rhythmicity may become uncoupled, favoring oxidative stress and reduced NO bioavailability (154). These alterations may weaken temporal vascular buffering capacity and contribute to oxidative stress-related endothelial glycocalyx injury (155). Glycocalyx injury, together with altered rhythmic expression of tight junction proteins such as Claudin-5 and ZO-1, may increase endothelial permeability and facilitate subendothelial low-density lipoprotein entry in susceptible vascular regions (156). In this setting, the most relevant circadian dimension is the timing of endothelial barrier function, leukocyte entry, lipid handling, and extracellular matrix repair within the vascular wall.

Endothelial barrier dysfunction may also influence the behavior of underlying vascular smooth muscle cells (VSMCs). The rhythmicity of VSMCs is central to maintaining the structural stability of the tunica media; the molecular clock maintains VSMCs in a highly differentiated contractile phenotype by repressing transcription factors such as KLF4 (157). When circadian regulation is impaired, this transcriptional restraint may be weakened, favoring a shift toward a less contractile and more synthetic phenotype in experimental models (158). These synthetic-like VSMCs may increase matrix metalloproteinase activity and disturb collagen and elastin turnover rhythms (159). Such changes may contribute to elastic fiber fragmentation, vascular calcification, and reduced biomechanical compliance of the arterial wall. This clock-driven matrix remodeling manifests macroscopically as increased pulse wave velocity (PWV) and microscopically as a thinning, brittle fibrous cap (160). If rhythmic repair programs are impaired, the vessel wall may become more susceptible to shear stress-related injury, which could contribute to plaque vulnerability in specific pathological contexts (156). Although time-of-day variation in acute coronary events is supported by clinical observations, direct evidence that circadian disruption independently causes plaque rupture remains limited. Circadian mechanisms should therefore be interpreted as potential modifiers of plaque vulnerability and thrombogenicity rather than as sole triggers of rupture.

### 4.3 Myocardial infarction and post-ischemic heart failure

**Figure 3:**
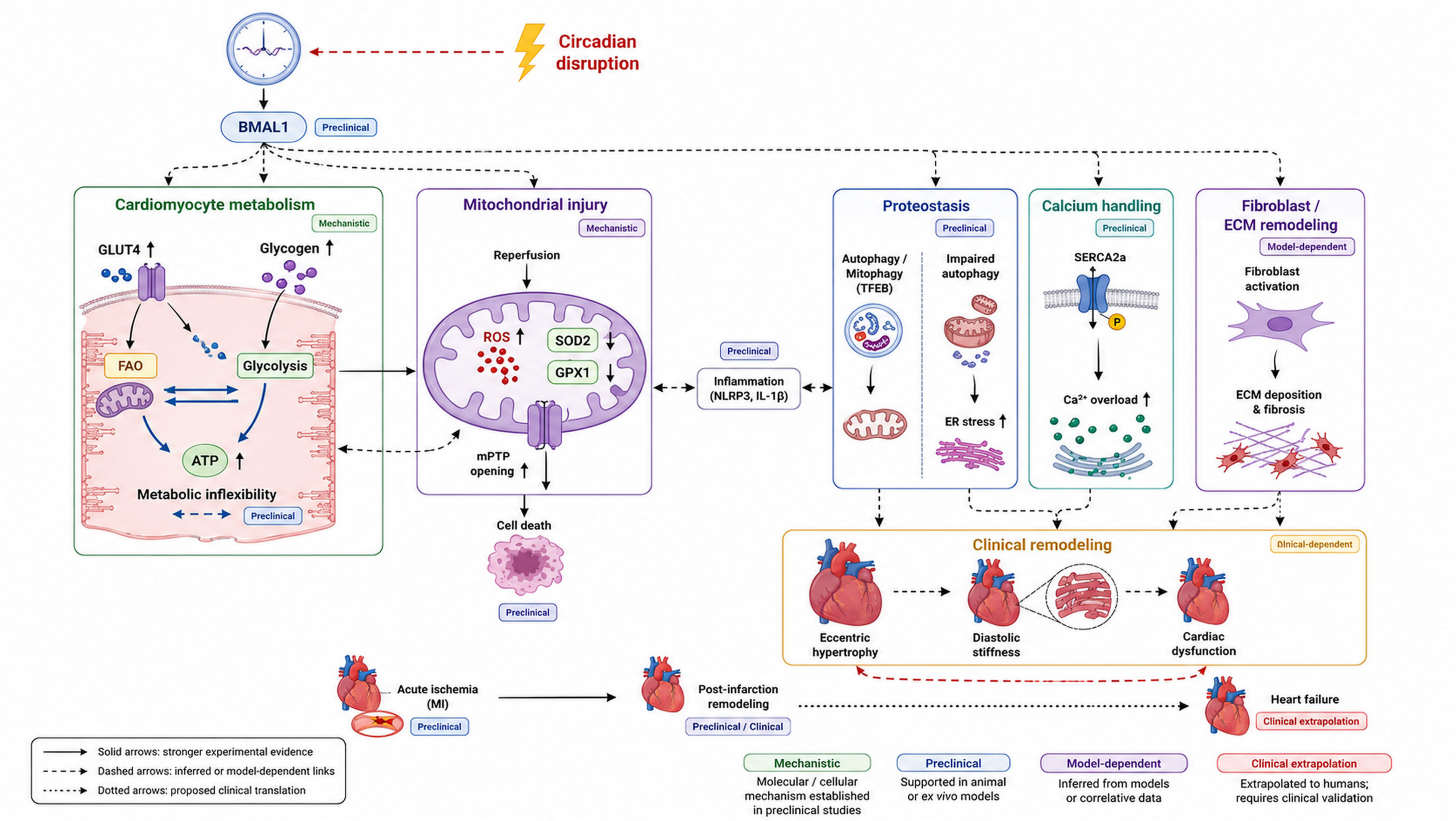
Circadian regulation of myocardial ischemia-reperfusion injury and adverse remodeling. This schematic summarizes proposed mechanisms through which disrupted circadian timing may influence myocardial ischemia-reperfusion injury and post-infarction remodeling. In experimental models, altered BMAL1-dependent metabolic regulation has been linked to changes in glucose utilization, glycogen availability, and mitochondrial stress responses. During reperfusion, impaired temporal coordination of antioxidant defenses may increase susceptibility to mitochondrial permeability transition pore opening and cardiomyocyte injury. In the chronic phase, disruption of autophagy, calcium handling, and fibroblast activation may contribute to adverse remodeling. These pathways should be interpreted as model-dependent mechanisms with varying levels of evidence rather than as a universal causal sequence.

Circadian timing may modulate the severity of myocardial ischemia-reperfusion injury and post-infarction repair, although the available evidence should be interpreted in light of species differences, experimental timing, and the distinction between intrinsic cardiac clocks and behavioral circadian disruption (161, 162). During the acute phase of myocardial infarction (MI), cardiac survival hinges on metabolic flexibility—the capacity to swiftly transition between fatty acid oxidation and glycolysis to meet hypoxic challenges (163). Experimental evidence suggests that cardiomyocyte-intrinsic clocks contribute to metabolic gating by regulating glucose handling, glycogen availability, and rhythmic expression of glucose transporters such as GLUT4 before the active phase (164). However, when these rhythms are disrupted, cardiomyocytes may lose metabolic preparedness and fail to efficiently upregulate glucose utilization during an ischemic insult (86) (**Figure 3**). This temporal mismatch may reduce metabolic flexibility and increase susceptibility to ATP depletion, acidosis, and larger infarct size in experimental models (86). Clinical observations have reported time-of-day differences in myocardial injury severity, particularly during the early morning hours, but these findings may be influenced by symptom onset, treatment delay, sleep status, medication use, and comorbidities (165, 166). When interpreting infarct-timing studies across species, rodent ZT should be mapped to rest-active phase rather than directly to human clock time. A rodent dark-phase event reflects the active phase of a nocturnal animal, whereas the clinically recognized early-morning vulnerability in humans occurs around the rest-to-active transition (**Figure 3**). One proposed mechanism involves desynchronization of mitochondrial redox defenses, in which clock-controlled antioxidant enzymes, such as SOD2 and GPX1, may fail to rise appropriately during reperfusion, thereby increasing susceptibility to mitochondrial permeability transition pore opening and cardiomyocyte injury (167). In myocardial ischemia-reperfusion injury, circadian timing is most relevant to cardiomyocyte metabolic readiness, mitochondrial redox defense, and post-infarction repair dynamics.

In the subsequent post-infarction repair phase, circadian desynchrony may disturb proteostasis and calcium signaling dynamics (168). As high-energy-demand cells, cardiomyocytes rely heavily on the rhythmic activation of autophagy and mitophagy, processes mediated by the transcription factor TFEB, to maintain functional integrity (169). Under circadian misalignment, the clearance of damaged mitochondria and misfolded proteins stalls, leading to the accumulation of dysfunctional organelles and persistent endoplasmic reticulum (ER) stress (163). Furthermore, the cardiac clock maintains calcium homeostasis by modulating the phosphorylation of SERCA2a (Sarcoplasmic Reticulum Calcium ATPase); its disruption impairs calcium reuptake, inducing intracellular calcium overload (170). This failure of intracellular “housekeeping” shifts the trajectory of post-MI remodeling from organized scar formation to maladaptive, eccentric hypertrophy (171). Concurrently, the inability of the cardiac clock to synchronize fibroblast activation results in disorganized collagen deposition and excessive fibrosis. Such structural “stiffening” markedly reduces diastolic compliance and exacerbates ventricular loading. Together, these findings support a model in which disrupted cardiac timing may lower the threshold for ischemic injury and impair coordinated repair after myocardial infarction. Nevertheless, this model remains most strongly supported by preclinical and mechanistic evidence, and its translation to human post-infarction remodeling requires cautious interpretation (172).

### 4.4 Arrhythmias and sudden cardiac death

Circadian timing is associated with daily variation in electrophysiological vulnerability and sudden cardiac death (SCD) risk. However, in human studies, the relative contributions of intrinsic ion-channel rhythms, autonomic tone, sleep disruption, medication timing, and comorbid cardiovascular disease are difficult to separate (141). The pacing and conduction properties of the atria, ventricles, and specialized conduction systems exhibit profound circadian rhythms, governed by the intrinsic molecular clock’s regulation of voltage-gated ion channels (e.g., Kv4.2, Nav1.5, HCN4) (173). In the context of circadian desynchrony, this coordinated ionic rhythm may be weakened, contributing to abnormal variation in action potential duration (APD) across the diurnal cycle in experimental models (174). Evidence highlights that the core clock protein BMAL1 directly regulates the transcription of *KLF15*, a master regulator of voltage-gated potassium channels involved in ventricular repolarization (175). Disruption of this axis may increase transmural dispersion of repolarization and lower the threshold for ventricular tachycardia or fibrillation in susceptible experimental or clinical contexts (141). For arrhythmias, the central issue is electrophysiological timing, including rhythmic regulation of ion channels, autonomic tone, conduction properties, and calcium handling, rather than vascular inflammation alone.

Beyond intrinsic ion-channel remodeling, autonomic imbalance associated with circadian disruption may contribute to temporal variation in SCD risk (176). While healthy rhythms provide electrophysiological protection through the balanced interplay of vagal and sympathetic drives, circadian misalignment often leads to an exaggerated sympathetic surge upon awakening (177). In an electrically vulnerable myocardium, an exaggerated sympathetic surge may promote calcium-handling abnormalities and triggered activity (178). Concurrently, gap junction proteins such as Connexin 43, whose expression and localization are rhythmically gated by clock genes, undergo downregulation and lateralization under circadian stress (179). This not only slows conduction velocity but also provides the structural substrate for reentry circuits. Together, these findings suggest that circadian timing may influence arrhythmic vulnerability through both cell-intrinsic electrophysiological pathways and systemic autonomic rhythms. However, the translation of these mechanisms to human arrhythmogenesis requires careful consideration of sleep quality, ischemic burden, electrolyte status, medication timing, and underlying structural heart disease.

### 4.5 Evidence strength across circadian-related cardiovascular pathologies

This subsection does not propose a common mechanism for all additional cardiovascular diseases; instead, it summarizes heterogeneous conditions according to the dominant type and strength of circadian evidence. Beyond hypertension, atherosclerosis, myocardial infarction, and arrhythmia, circadian mechanisms have been implicated in several additional cardiovascular conditions, but the strength of evidence is uneven across disease contexts (**Table 2**). Some associations, such as time-of-day variation in thrombotic tendency, are supported by human temporal patterns together with mechanistic evidence from coagulation and fibrinolytic biology (180). In contrast, links between circadian disruption and pulmonary arterial hypertension, aneurysm progression or rupture, valvular degeneration, peripheral artery disease, and chronic heart failure are supported to varying degrees by preclinical models, tissue-specific clock perturbation, associative clinical observations, or mechanistic extrapolation from related pathways (181). Therefore, these conditions should not be presented as equivalent clinical consequences of circadian disruption. To provide a more evidence-weighted interpretation, **Table 2** grades each condition according to the dominant type of supporting evidence and distinguishes relatively stronger clinical evidence from limited animal data or hypothesis-generating mechanistic inference.

**Table 2:** Evidence-graded summary of additional circadian-related cardiovascular pathologies.

Disease Type	Proposed circadian-related mechanisms	Clinical/pathological association	Evidence level	Interpretation	References
Pulmonary arterial hypertension (PAH)	BMAL1-related signaling has been linked to BMX kinase activation, PPARγ downregulation, and pulmonary vascular remodeling in experimental settings.	Nocturnal pulmonary pressure variation and right ventricular stress may be influenced by circadian and autonomic regulation.	Limited preclinical/mechanistic evidence	Suggestive but insufficient to establish circadian disruption as a direct driver of human PAH progression.	(182)
Deep vein thrombosis (DVT)	CLOCK/BMAL1-dependent regulation of PAI-1 may contribute to time-of-day variation in fibrinolytic activity.	Morning hypercoagulability and temporal variation in thrombotic events have been reported.	Relatively strong clinical + mechanistic evidence	Supported by human temporal patterns and coagulation biology, although individual risk is modified by immobility, hospitalization, comorbidities, and medication use.	(183)
Abdominal aortic aneurysm (AAA)	REV-ERBα-related pathways may regulate inflammatory and matrix-degrading programs, including MMP activity, in experimental models.	Circadian patterns of rupture have been suggested, but disease-specific human evidence remains limited.	Limited preclinical/mechanistic evidence	Hypothesis-generating; direct evidence linking circadian disruption to human aneurysm expansion or rupture remains limited.	(151)
Valvular heart disease	Clock-related pathways may interact with KLF15-Notch1 signaling and osteogenic programs in valvular interstitial cells.	Possible association with valvular degeneration, but direct clinical timing evidence is sparse.	Hypothesis-generating extrapolation	Mechanistically plausible but requires disease-specific validation in human valvular degeneration.	(184)
Peripheral artery disease (PAD)	PER2-related metabolic regulation may influence VEGF signaling, angiogenesis, and ischemic tissue repair.	Symptoms may vary with activity and vascular demand, but circadian-specific human evidence is limited.	Limited preclinical/mechanistic evidence	Potentially relevant to repair biology, but clinical circadian evidence remains weak.	(185)
Chronic heart failure (CHF)	NR1D1-related pathways may influence natriuretic peptide signaling, volume regulation, autonomic tone, and myocardial metabolism.	Nocturnal symptoms and volume shifts are clinically recognized, but direct circadian-causal evidence is limited.	Moderate associative + mechanistic evidence	Circadian mechanisms may contribute to symptom timing and neurohormonal regulation, but are not established as primary drivers of CHF progression.	(100)

Overall, this grading highlights that circadian biology is most strongly supported as a modifier of temporal cardiovascular risk in conditions with clear daily event patterns, whereas its role in several structural cardiovascular diseases remains mechanistically plausible but incompletely validated in humans (**Table 2**).

## 5 Circadian immune mechanisms linking timing disruption to vascular and myocardial pathology

Whereas Section 3 describes physiological immune rhythmicity, this section focuses on selected pathological immune mechanisms through which disrupted timing may contribute to vascular and myocardial disease. The emphasis is placed on mechanistic specificity rather than repeating the general sequence of clock disruption, inflammation, and injury.

### 5.1 Network-based epigenetic gating of pro-inflammatory enhancers in myeloid cells

The pathogenicity of myeloid cells, such as monocytes and macrophages, in cardiovascular inflammation is determined not only by the intensity of external stimuli but also by the rhythmic fluctuations in chromatin accessibility (186). REV-ERBα is one component of an epigenetic timing network that helps shape immune-response thresholds through rhythmic enhancer occupancy and co-repressor recruitment (187). At the molecular level, REV-ERBα can recruit the Nuclear Receptor Co-repressor (NCoR) and Histone Deacetylase 3 (HDAC3) to regulatory sites of selected pro-inflammatory genes, including *Ccl2, Il6*, and *Tnf (*188). This complex can promote H3K27 deacetylation and reduce local chromatin accessibility at selected inflammatory regulatory regions (189). This regulatory state may limit access of NF-κB and co-activators such as p300/CBP to selected enhancers, thereby helping restrain inflammatory gene expression during specific phases (190).

Furthermore, recent evidence suggests that this epigenetic gating extends beyond mature circulating cells to the early programming stages of hematopoietic stem and progenitor cells (HSPCs) in the bone marrow (191). Driven by circadian misalignment, the epigenetic landscape within HSPCs undergoes characteristic shifts, manifested by rhythmic aberrations in histone modifications at the promoters of myeloid differentiation factors, such as PU.1 (192). Such changes may bias hematopoietic output toward monocytes with heightened inflammatory potential, although the durability and disease specificity of this effect require further validation (192). Altered REV-ERBα-HDAC3 signaling may also interact with immunometabolic rhythms (188). Under normal rhythms, clock genes gate the metabolic threshold for the transition from oxidative phosphorylation to glycolysis in macrophages; however, upon the withdrawal of this gating, heightened chromatin accessibility allows for the over-expression of key metabolic enzymes, essentially pre-programming cells for pro-inflammatory behaviors before they even infiltrate the damaged vasculature (193).

When endogenous rhythms are disrupted or phase-shifted, this epigenetic homeostasis may become unstable (194). Enhancers previously under temporal control remain in a state of sustained H3K27 hyperacetylation, trapping the chromatin in a constitutively open conformation (195). This sustained accessibility not only lowers the biological threshold for inflammatory activation but also significantly increases transcriptional noise, causing myeloid cells to persistently “leak” pro-inflammatory signals even in the absence of canonical pathological triggers (195). Additionally, the reorganization of chromatin looping mediated by long non-coding RNAs (lncRNAs) plays a pivotal role (196). In the context of circadian misalignment, the loss of temporal gating by REV-ERBA allows distal enhancers to remain physically coupled with promoters of core pro-inflammatory genes (197). This transition from rhythmic repression toward sustained enhancer accessibility may promote a primed inflammatory state in infiltrating monocytes and contribute to immune-mediated vascular injury in susceptible contexts.

Thus, the epigenetic control of myeloid inflammation should be understood as a distributed network involving REV-ERBα-HDAC3 repression, BMAL1:CLOCK-dependent transcriptional timing, PER/CRY feedback, ROR-mediated activation, metabolic cofactors, and tissue-derived inflammatory cues (17, 18). This network-based view avoids reducing myeloid pathogenicity to failure of a single clock component and better reflects the compartment-specific nature of cardiovascular inflammation.

### 5.2 Circadian gating of leukocyte trafficking and vascular recruitment

The distribution of immune cells between the circulation and peripheral tissues is not entirely stochastic but is partly shaped by circadian rhythms (47). The fundamental logic of this rhythmicity is to synchronize maximal immunosurveillance with the active phase, while facilitating the recession of leukocytes to the bone marrow or secondary lymphoid organs for homeostatic maintenance during the rest phase (198). At the molecular level, the efficiency of leukocyte egress from the bone marrow and subsequent attachment to the vascular intima is heavily contingent upon the temporal coupling of the CXCL12/CXCR4 chemokine axis (89, 199). Under physiological conditions, endogenous bone marrow clocks contribute to rhythmic CXCL12 expression and help regulate daily variation in circulating leukocyte availability (200). In the context of circadian misalignment, disruption of this timing system may disturb leukocyte retention, egress, and endothelial interaction, thereby favoring inappropriate vascular recruitment (198). This spatiotemporal mismatch may increase inflammatory burden within the vessel wall and disturb vascular immune quiescence.

Deeper immunodynamic investigations reveal that circulating neutrophils undergo a life cycle of “aging” strictly monitored by their cell-intrinsic clocks (201). In a normal physiological sequence, as neutrophils prolong their tenure in circulation, they exhibit characteristic phenotypic drifts, specifically the polar up-regulation of the pro-migratory receptor CXCR4 and the gradual down-regulation of the adhesion molecule L-selectin (CD62L) (87). This aging process, driven by rhythmic redox homeostasis, undergoes significant phenotypic polarization under circadian desynchrony, resulting in an abnormally high proportion of “hyper-inflammatory” aged neutrophils in the peripheral blood. These cells possess a heightened potential for reactive oxygen species (ROS) production and a greater propensity for the uncontrolled release of neutrophil extracellular traps (NETs) (47). Concurrently, the “capture window” of the vascular intima is governed by the phase of endothelial cell clocks (202). Endothelial cells dictate the accessibility of the vessel wall to immune cells by regulating the expression phases of surface adhesion molecules, such as E-selectin and VCAM-1 (203). When circadian disruption causes phase shifts or constitutive openness of the endothelial barrier, abnormally aged immune cells can easily transmigrate even during the rest phase (204).Together, chemokine-axis dysregulation, altered neutrophil aging, and endothelial adhesion changes may convert physiological leukocyte recruitment into sustained vascular inflammation. However, whether these mechanisms directly trigger acute cardiovascular events in humans remains uncertain.

### 5.3 Transcriptional gating of immune polarization and subset bias

The outcome of cardiovascular tissue following injury is influenced by the local balance of immune subsets, which may be shaped by clock-dependent variation in transcriptional programs (205).In macrophages, clock-related pathways may modulate responsiveness to inflammatory signals and influence transitions among pro-inflammatory, reparative, and mixed activation states, partly through transcription factors such as KLF4 (206). Under physiological timing, such polarization programs may support an early inflammatory response for debris clearance and later contribute to reparative signaling during tissue healing (207). However, circadian misalignment may impair this phenotypic transition and prolong pro-inflammatory transcriptional programs, including NF-κB-related signaling, in susceptible tissues (208). This may favor persistence of inflammatory macrophage states in post-infarct myocardium or atherosclerotic plaques, although the magnitude and direction of this effect are likely context-dependent. This clock-associated phenotypic bias may shift tissue repair toward maladaptive remodeling and contribute to chronic heart failure progression.

Rhythmic features of the adaptive immune system may further influence tissue inflammation and repair (209). The differentiation of T-lymphocyte subsets, particularly the balance between Th1/Th17 cells and regulatory T cells, may be influenced by clock-dependent variation in cytokine receptor sensitivity and tissue-specific inflammatory cues (125).Under physiological conditions, this equilibrium ensures that the immune response does not exert excessive aggression against self-tissues. In the context of circadian dysfunction, Th17-associated responses may be favored and Treg-mediated restraint may be weakened, although this balance is likely to vary by disease stage, tissue context, and experimental model (210). This “pathogenic drift” in adaptive immunity not only exacerbates chronic autoimmune attacks on the vessel wall but also induces diffuse myocardial fibrosis and loss of vascular elasticity by altering the residency dynamics of lymphocytes within damaged tissues (119). This shift in immune-cell state suggests that disrupted transcriptional timing may convert normally protective immune responses into sustained inflammatory programs that impair vascular and myocardial homeostasis.

### 5.4 Circadian integration of inflammasome activation and pyroptosis

The NLRP3 inflammasome represents an important effector pathway linking inflammatory signaling to cardiovascular injury and is influenced by circadian regulatory mechanisms (211). Under physiological conditions, core clock proteins, such as BMAL1 and REV-ERBA, strictly limit the basal expression of NLRP3 and its adaptor protein ASC through transcriptional repression, thereby setting the threshold for the “priming signal” of inflammatory ignition (212). This rhythmic suppression may help limit inappropriate inflammatory activation in cardiomyocytes and vascular endothelial cells in response to physiological stressors, such as normal blood pressure fluctuations or minor metabolic shifts. However, upon circadian disruption, this molecular barrier constructed by clock genes rapidly collapses, leading to a pathological over-expression of NLRP3 protein and rendering the inflammasome hypersensitive to danger-associated molecular patterns (DAMPs) without any phase constraints (213).

The more pathologically significant mechanism lies in the circadian integration of the “activation signal” for the inflammasome. Within a chronobiologically intact cardiovascular microenvironment, NR1D1 (REV-ERBA) prevents the final assembly of the NLRP3 complex by inhibiting specific signaling pathways, such as the phosphorylation of TLR4/NF-κB (214). Once circadian desynchronization occurs, this temporal lock is released, causing cardiomyocytes to undergo uncontrolled inflammasome polymerization in response to minor ischemia or cholesterol crystal stimuli (215). This explosive activation not only leads to the massive maturation and release of pro-inflammatory cytokines IL-1B and IL-18 but also directly triggers pyroptosis (216). This is a programmed cell death mode mediated by Gasdermin D (GSDMD), characterized by the formation of membrane pores and the instantaneous expulsion of cellular contents. In the pathological context of myocardial infarction or atherosclerosis, pyroptosis induced by the failure of circadian regulation amplifies local microscopic immune responses into tissue-scale destruction, eventually converting inflammatory burdens into irreversible pump failure and vascular structural collapse, thereby elucidating the terminal molecular logic of circadian disruption as an “accelerator” of lethal cardiovascular events (170).

### 5.5 Integrative chrono-immunology and microenvironmental synergism

The progression of cardiovascular pathology is not an isolated act of a single immune cell type but rather the result of synergistic dysregulation among multiple cellular subpopulations within the temporal dimension (217). The biological clock mediates “paracrine coupling” between immune cells and inherent vascular cells (endothelial and smooth muscle cells), constructing a dynamic immune microenvironment (218). Under normal physiological conditions, signaling molecules released by endothelial cells, such as nitric oxide (NO) and prostacyclin, exhibit distinct temporal phases (219). These molecules do not only regulate vascular tone but also function as anti-inflammatory signals, exerting a “negative feedback” modulation during the leukocyte recruitment window. However, endothelial clock dysfunction induced by circadian misalignment leads to a temporal deficit of these protective signals (220). Concurrently, smooth muscle cells, suffering from clock-regulated impairment, actively attract and sequester “primed” monocytes by secreting aberrant levels of chemokines (e.g., MCP-1) and extracellular matrix remodeling enzymes. This clock-driven signaling misinformation initiated by non-immune cells distorts reparative signals into sustained recruitment commands, turning the vascular wall into an uncontrolled immunological “battleground”.

Deeper integrative mechanisms involve the “neuro-immune rhythmic axis” between the immune system and the autonomic nervous system. The release of neurotransmitters by sympathetic nerve fibers within cardiovascular tissues exhibits a significant circadian rhythm; these signals directly modulate the inflammatory cytokine release thresholds via adrenergic receptors on the surface of leukocytes (221). In the context of circadian desynchronization, the phase coupling between nerve endings and immune cells is severed, causing the immune system’s “stress response” to sympathetic activation to lose its temporal constraints (222).This multidimensional disruption of rhythmic interactions not only explains the molecular basis of why immune-mediated cardiovascular injury exhibits a pronounced morning peak but also reveals how circadian misalignment amplifies minor molecular shifts into systemic pathological disasters by reshaping the “temporal microenvironment” of the entire organ (223). This integrative chrono-immunological perspective provides a critical theoretical foundation for the development of cardiovascular immunotherapies targeting biological clock components.

## 6 Chronopharmacology in cardiovascular immune modulation: evidence, controversies, and translational barriers

Chronopharmacology provides an attractive framework for aligning drug exposure with daily variation in blood pressure, platelet activity, lipid metabolism, inflammatory tone, and immune-cell trafficking (33). However, its clinical application in cardiovascular disease remains at an early and uneven stage. The available evidence differs substantially across drug classes: antihypertensive chronotherapy has been tested in large outcome trials but remains controversial; statin timing is supported mainly by pharmacokinetic and lipid-lowering data; antiplatelet timing has shown effects on platelet reactivity but has limited outcome-level validation; and anti-inflammatory or immunomodulatory chronotherapy remains largely mechanistic or preclinical (224). Therefore, time-of-day dosing should be considered a context-dependent strategy rather than a universally applicable therapeutic principle (225). Therapeutic windows derived from rodent ZT experiments require phase-based translation before clinical application. A dosing time that targets the rodent active phase should not be directly assigned to the same clock hour in humans, but should be reconsidered in relation to human wake time, sleep timing, chronotype, and measurable circadian phase markers (224).

### 6.1 Evidence across major cardiovascular drug classes

Among antihypertensive agents, bedtime dosing has been proposed to improve nocturnal blood pressure control and attenuate the morning blood pressure surge. Nevertheless, clinical findings have been inconsistent (226). While earlier studies suggested potential cardiovascular benefit from bedtime dosing, later large randomized evidence, including the TIME study, found no significant difference in major cardiovascular outcomes between evening and morning dosing of usual antihypertensive therapy (227). These findings suggest that antihypertensive timing should not be generalized across all patients and should consider drug half-life, nocturnal hypotension risk, tolerability, and adherence.

For antiplatelet therapy, circadian variation in platelet reactivity provides a plausible rationale for time-of-day dosing. Bedtime administration of low-dose aspirin has been reported to reduce morning platelet reactivity in some studies (228). However, most available data focus on pharmacodynamic markers rather than hard cardiovascular outcomes. Therefore, antiplatelet chronotherapy remains biologically plausible but clinically insufficiently established, particularly in acute coronary syndromes where rapid administration and guideline-based treatment take priority over circadian optimization (229). Statins provide a different example because timing effects are closely related to drug half-life and hepatic cholesterol synthesis (230). Evening dosing may be more relevant for short-acting statins, whereas long-acting statins can often be administered at a time that improves adherence. Current evidence therefore supports a pragmatic interpretation: timing may matter for selected statins and lipid endpoints, but the clinical importance of timing is likely smaller than treatment intensity, drug selection, and long-term adherence (231).

Compared with antihypertensive, antiplatelet, and statin therapy, chronotherapy for anti-inflammatory and immunomodulatory interventions in cardiovascular disease remains less mature (232). Circadian regulation of the NLRP3 inflammasome, IL-1β signaling, leukocyte recruitment, and macrophage polarization provides a strong mechanistic rationale, but most evidence comes from experimental models or biomarker-based studies (33). At present, there is insufficient clinical evidence to recommend routine time-of-day dosing for cardiovascular anti-inflammatory or immune-targeted therapies. Future trials should test whether circadian phase-guided administration improves efficacy or safety beyond standard dosing schedules (233).

### 6.2 Experimental clock-targeted and immune-modulatory approaches

Clock-targeted and immune-modulatory approaches represent a more experimental layer of cardiovascular chronopharmacology. Small-molecule agonists targeting core clock-related proteins such as REV-ERBα and RORα have shown potential in experimental settings to reshape immune-cell epigenetic programs, but these strategies remain far from routine cardiovascular clinical use (234). In principle, phase-selective activation of these pathways could reinforce endogenous anti-inflammatory programs and reduce chemokine transcription, including Ccl2, during vulnerable immune phases (200). Similarly, interventions targeting the CXCL12/CXCR4 axis may help clarify whether timed modulation of leukocyte egress can reduce maladaptive vascular recruitment (89). However, these approaches should currently be interpreted as hypothesis-generating or preclinical rather than as validated therapeutic strategies. Rather than blindly suppressing the immune system long-term, this strategy seeks to repair the spatial distribution logic of immune cells, preserving normal immunosurveillance functions (235).

In acute cardiovascular settings, the feasibility of chronotherapy is more limited. Although preclinical studies suggest that inflammatory pathways such as the NLRP3 inflammasome and IL-1β signaling may exhibit time-of-day-dependent activity during myocardial ischemia-reperfusion injury, acute care is primarily determined by symptom onset, reperfusion timing, hemodynamic stability, and guideline-directed emergency treatment (5). Therefore, phase-synchronized anti-inflammatory therapy is mechanistically attractive but cannot override the clinical priority of rapid diagnosis and treatment (**Table 3**). Such a strategy may reduce inflammatory amplification and support reparative immune transitions in experimental models, but whether similar benefits can be achieved safely in patients remains unknown. Non-pharmacological approaches, including light exposure management and time-restricted feeding, are also being explored as ways to modify systemic circadian alignment (74). However, their cardiovascular benefits, safety, adherence, and interaction with pharmacological therapy require further evaluation (236). Overall, chronopharmacology should currently be framed as an emerging precision-medicine concept with selected supportive data, substantial uncertainties, and a need for prospective trials that incorporate circadian phase assessment, chronotype, sex, age, comorbidity burden, and adherence (**Table 3**).

**Table 3:** Evidence and translational considerations for time-of-day dosing in cardiovascular therapy.

Drug class	Chronobiological rationale	Current evidence	Main limitations	Practical interpretation	References
Antihypertensives	Blood pressure, sympathetic tone, and renin–angiotensin activity show daily variation	Large trials have tested morning versus evening dosing, but cardiovascular outcome benefits remain inconsistent	Risk of nocturnal hypotension, drug half-life differences, chronotype variability, adherence	Timing should be individualized; universal bedtime dosing is not supported	(237)
Antiplatelet agents	Platelet reactivity and thrombotic risk often peak in the morning	Bedtime aspirin may reduce morning platelet reactivity in some studies	Mostly pharmacodynamic endpoints; limited outcome-level evidence	Biologically plausible but not established for routine outcome-based chronotherapy	(238)
Statins	Hepatic cholesterol synthesis is more active at night	Evening dosing may benefit short-acting statins; long-acting statins can often be taken flexibly	Lipid endpoints dominate; outcome-level timing evidence is limited	Timing may matter for short-acting statins, but adherence remains central	(239)
Anti-inflammatory therapies	Cytokine release, inflammasome activity, and immune activation thresholds may be rhythmic	Mainly preclinical or biomarker-based evidence	Lack of large cardiovascular trials testing dosing time	Promising but investigational	(240)
Immunomodulators/clock-targeted agents	Clock proteins and leukocyte trafficking pathways regulate immune timing	REV-ERBα/RORα and CXCL12/CXCR4 strategies are mostly experimental	Safety, target specificity, phase assessment, clinical feasibility	Mechanistic concept requiring clinical validation	(241)

## 7 Limitations and translational challenges

Several limitations should be considered when interpreting the current evidence linking circadian immune regulation to cardiovascular disease. First, circadian measurement remains heterogeneous across studies. Human investigations have used diverse proxies, including clock time, sleep–wake timing, chronotype questionnaires, shift-work status, actigraphy, melatonin onset, cortisol rhythms, body temperature, and time-stamped clinical events. These approaches do not measure the same biological construct. Clock time alone may not reflect internal circadian phase, particularly in individuals with irregular sleep schedules, shift work, social jetlag, or metabolic disease. This heterogeneity makes it difficult to compare studies directly or to define universal “morning,” “night,” “rest-phase,” or “active-phase” risk windows.

Second, much of the mechanistic evidence comes from preclinical models, especially nocturnal rodents, genetic clock disruption, controlled light–dark manipulation, or simulated circadian misalignment. These models are valuable for identifying causal pathways, but they do not fully reproduce the complexity of human cardiovascular disease. Species differences in rest–activity phase, immune-cell composition, metabolic rate, vascular biology, and lifespan may influence the direction and magnitude of circadian effects. Moreover, genetic deletion of core clock components may produce more severe and less physiological disruption than the partial, chronic, and behaviorally mediated circadian misalignment commonly observed in humans.

Third, observational studies of shift work, sleep loss, social jetlag, and light exposure at night are vulnerable to confounding. Circadian disruption in human populations often coexists with altered sleep duration, irregular meals, psychosocial stress, socioeconomic factors, obesity, hypertension, diabetes, physical inactivity, smoking, and medication timing. These factors may independently affect cardiovascular risk and inflammatory tone. Therefore, associations between behavioral circadian disruption and cardiovascular outcomes should not be interpreted as direct causal evidence unless supported by careful adjustment, longitudinal design, objective circadian measurements, or intervention data.

Fourth, the clinical trial base for cardiovascular chronotherapy remains limited and uneven across drug classes. Although time-of-day dosing has been studied for antihypertensive agents, statins, antiplatelet therapy, and selected anti-inflammatory strategies, evidence for hard cardiovascular outcomes remains inconsistent or incomplete. Large prospective trials that incorporate circadian phase assessment, chronotype, sex, age, comorbidity burden, medication adherence, and safety endpoints are still needed. In acute cardiovascular settings, such as myocardial infarction or stroke, rapid diagnosis and guideline-directed treatment remain the priority, and phase-synchronized therapy may be difficult to implement without delaying care.

Fifth, inter-individual biological variability poses a major translational challenge. Chronotype, sex, aging, endocrine status, occupational schedule, comorbidities, and medication use can alter circadian amplitude, phase stability, and immune rhythmicity. Older adults may exhibit attenuated rhythm amplitude, while sex hormones may influence clock-gene expression, immune responses, vascular tone, and inflammatory thresholds. These factors suggest that a single clock-time-based dosing strategy is unlikely to be appropriate for all patients.

Finally, translating molecular phase into clinical decision-making remains unresolved. Many mechanistic studies define phase using tissue-specific gene expression, chromatin accessibility, or metabolic oscillations, whereas routine clinical practice relies on clock time, symptom onset, and medication schedules. Reliable, minimally invasive biomarkers of internal circadian phase are not yet widely available in cardiovascular care. Future studies should integrate wearable monitoring, serial biomarker sampling, multi-omics profiling, and pragmatic clinical trial designs to determine whether circadian phase-guided prevention or treatment can improve outcomes beyond standard risk-based care.

## 8 Conclusions and future perspectives

In conclusion, circadian regulation represents an important temporal layer in cardiovascular immune homeostasis. This review summarizes evidence that biological clocks help establish thresholds for inflammatory activation, contribute to rhythmic leukocyte trafficking, and may influence macrophage polarization and inflammasome-related responses through transcriptional, epigenetic, metabolic, and neuroendocrine mechanisms (242). Rather than defining cardiovascular disease as a direct consequence of failed “temporal gating,” we propose that disrupted circadian organization may act as a context-dependent modifier of immune activation, vascular vulnerability, and myocardial repair (243). This evidence-weighted perspective distinguishes established molecular mechanisms from model-dependent observations and emerging hypotheses, and may help guide future studies of cardiovascular inflammation.

Despite growing mechanistic insight, translation into clinical practice remains challenging. Much of the current evidence relies on nocturnal rodent models, genetic clock disruption, or simulated circadian misalignment, and these findings cannot be directly extrapolated to human cardiovascular disease without considering species differences, behavioral exposures, sleep status, comorbidities, and medication timing. Inter-individual variation in chronotype, sex, age, endocrine status, occupational schedule, and circadian phase further complicates standardized chronotherapeutic protocols (244). Future studies should integrate wearable monitoring, serial biomarker sampling, multi-omics profiling, and pragmatic clinical trial designs to determine whether circadian phase-guided prevention or treatment can improve outcomes beyond standard risk-based care.

Several questions remain unresolved. For example, the durability of “rhythmic memory” in hematopoietic stem and progenitor cells, the influence of sex-related clock regulation on immune responses, and the clinical utility of internal phase biomarkers require further investigation (245). Future cardiovascular prevention and treatment may increasingly incorporate chronobiological principles alongside established management of lipids, blood pressure, inflammation, and lifestyle risk factors. At present, however, chronopharmacology should be viewed as a promising but still developing framework rather than a validated replacement for guideline-directed cardiovascular care.
